# The effect of partial substitution of noble metal (Pd) at the B site of La0.6Sr0.4FeO3 as a supercapacitor electrode material

**DOI:** 10.55730/1300-0527.3639

**Published:** 2023-11-13

**Authors:** Nagihan DELİBAŞ, Turgut SÜLEYMANOĞLU

**Affiliations:** Department of Physics, Faculty of Science, University of Sakarya, Sakarya, Turkiye

**Keywords:** Symmetric supercapacitor, electrode material, partial substitution, perovskite oxide, electrochemical performance

## Abstract

Sol-gel combustion was used to produce the perovskite-type La_0.6_Sr_0.4_FeO_3_ (LSF) and La_0.6_Sr_0.4_Fe_0.9_Pd_0.1_O_3_ (LSFP) materials and assessed as supercapacitor electrodes. The synthesized materials’ crystal structure, morphology, and electrochemical performance were thoroughly analyzed. The partial substitution of Pd in the B site of the LSF structure affected the electrochemical properties of this compound and improved its performance. In fact, the greatest effect of Pd substitution was on the content of oxygen vacancies, which are known as the active sites of the perovskite surface in the supercapacitor cell. The specific capacitance obtained for the sample containing Pd was about 80 F.g^−1^ at a current density of 1 A.g^−1^ in 1M KOH. In addition, this sample had a decreased intrinsic resistance to ion and electron diffusion. The remarkable structural and morphological features of LSFP contribute to its superior electrochemical performance. At a power density of 1000 W.kg^−1^ and a current density of 1 A.g^−1^, an LSFP symmetrical cell had an energy density of 44.45 W.h.kg^−1^.

## 1. Introduction

The increase in environmental pollution and the decrease in nonrenewable energy sources, followed by the increase in energy demand in the last few decades, made the search for energy production from renewable energy sources and efficient technologies in the field of energy production and energy storage to be very important [1–3]. Lithium-ion batteries are among the well-known energy storage systems used in almost all electronic devices today. But they have limited power density. Ordinary dielectric capacitors, unlike lithium-ion batteries, have a high power density but a very low energy density. Therefore, the existing gap between the lithium-ion battery and the conventional dielectric capacitor has community researchers to look for better alternatives for energy storage. Therefore, the gap between the lithium-ion battery and the conventional dielectric capacitor has prompted researchers to explore better alternatives for energy storage [4–6]. Supercapacitors are new electrochemical energy storage technologies that have the potential to bridge the gap between the preceding two systems. There are two different kinds of supercapacitors: pseudo-capacitors, which store energy by redox reactions of electrode materials during the charge-discharge process, and electrochemical double layer capacitors (EDLC), which store energy by electrostatic interaction [7,8]. Their wide applications in electric hybrid vehicles, portable devices, start and stop systems, etc. have made the supercapacitors known as a reliable energy storage system [9,10].

The electrode material is the most important part of the pseudo-capacitors structure because the electrochemical reaction takes place in this part. Carbon materials, metal oxides, conductive polymers, and perovskite oxides have been widely investigated as efficient electrode materials by many researchers [11–14].

Perovskite oxide (ABO_3_) electrode materials have attracted much attention due to their high specific capacitance, wide potential window, and excellent life cycle. The important feature of these compounds, which can insert ions into the materials’ bulk structure, is that they provide a high specific capacitance within a short period [15,16]. Perovskite oxides have two metal cation sites, and usually, the ionic radius of A site cation is larger than that of B site cation. Cations of both A and B sites can be substituted by other metal ions with different valences or ionic radii [1,7]. Substitution of different cations in A and B sites can affect the type and content of perovskite oxide structural defects, especially oxygen vacancies, and change its physicochemical properties. The oxygen vacancy of the structure as a charge carrier plays a key role in determining the mixed ionic and electronic conductivity (MIEC) of perovskite oxide. The structural tunability of perovskite, taking into account the Goldschmidt tolerance factor, provides a wide range of cationic substitutions in both A and B sites, which leads to improved electrochemical properties of perovskite oxide [16,17]. The effect of substitution of cations with different valences in A and B sites on the structure properties and electrochemical functionality of perovskite oxides has been widely investigated in previous articles. In 2014, for the first time, Mefford et al. proposed an anion-intercalated electrochemical energy storage mechanism for perovskite oxide supercapacitor electrodes [18]. Tomar et al. [19] substituted Mo at the B site of SrCoO_3_ perovskite oxide structure, and as a result, improved the oxygen vacancy content and increased the specific capacitance to 1224.34 F.g^−1^. Lang et al. [10] by substituting Sr^2+^ at A site of LaMnO_3_, were able to impose more distortion on the perovskite oxide structure and increase the content of oxygen vacancies, so that at a current density of 1 A.g^−1^, a specific capacitance of 102 F.g^−1^ was obtained for La_0.85_Sr_0.15_MnO_3_. According to studies, substituting high-valence cations in the B-site increases the oxygen vacancy concentration; additionally, substituting in the A site enhances the oxidation states of B site cations and creates more oxygen vacancies in the structure of perovskite oxides.

In this study, the electrochemical and structural properties of perovskite oxides La_0.6_Sr_0.4_FeO_3_ (LSF) and La_0.6_Sr_0.4_Fe_0.9_Pd_0.1_O_3_ (LSFP) have been investigated as supercapacitor electrode material. These two perovskite oxides have not before been studied as supercapacitor electrodes, as far as we know. Our aim of this research is to investigate the effect of Pd cation substitution in B site of La_0.6_Sr_0.4_FeO_3_ on its electrochemical properties. The electrochemical activity of the La_0.6_Sr_0.4_FeO_3_ and La_0.6_Sr_0.4_Fe_0.9_Pd_0.1_O_3_ was studied by cyclic voltammetric (CV) and galvanostatic charge-discharge (GCD). Also, the materials’ resistance to ion and electron emission, was evaluated by electrochemical impedance spectroscopy (EIS) analyses.

## 2. Experimental

### 2.1. Synthesis method and characterization

Lanthanium (III) nitrate hexahydrate (La(NO_3_)_2_ .6H_2_O, assay 98%>), Strontium nitrate (Sr(NO_3_)_2_, assay 98.0%), Iron (III) nitrate nonahydrate (Fe(NO_3_)_3_ .9H_2_O, assay 99%), Palladium (II) nitrate dehydrate (Pd(NO_3_)_2_ .2H_2_O, assay ~40% Pd basis) and Glycine (H_2_NCH_2_COOH, assay 99.0%, SAMCHUN, South Korea) were used as raw materials. Sol-gel combustion was the method used to prepare La_0.6_Sr_0.4_FeO_3_ and La_0.6_Sr_0.4_Fe_0.9_Pd_0.1_O_3_. The raw materials of perovskite oxides (La(NO_3_)_2_ .6H_2_O, Sr(NO_3_)_2_, Fe(NO_3_)_3_ .9H_2_O and Pd(NO_3_)_2_ .2H_2_O) were mixed in 50 mL of deionized water under heating in a stoichiometric ratio. When the temperature reached 60 °C, glycine was added to the above solution as an ignition agent. After that, heating and stirring were continued until a gel was formed. After the formation of the gel, heating was continued until the ignition of the gel. The combustion product was calcined at 550 °C for 4 h.

The crystal structure of the synthesized samples was investigated using X-ray diffraction (Tongda TD-3700, China). The resulting XRD spectrum was recorded at 2θ between 10° and 80°. Scanning electron microscopy (FE-SEM, model MIRA3-TESCAN, Czech) was employed to study and characterise the morphology of LSF and LSFP. FT-IR analysis (TENSOR 27, Brucker, Germany), energy dispersive X-ray spectroscopy (EDX, MIRA3 FEG-SEM, Tescan, Czech Republic), and elemental mapping were used to additional investigate the structure of perovskite oxides.

### 2.2. Electrochemical section

Electrochemical properties of synthesized perovskite oxides were investigated in a two-electrode system at room temperature. In this study, 1M KOH was used as an electrolyte. The electrochemical system is symmetrical and the anode and cathode of this system are made of synthesized perovskites. The electrodes included 3 mg of active materials (perovskite oxide 80 wt.%, activated carbon 15 wt.%, and Nafion solution 5 wt.%) which were coated on copper plates as current collectors. Cyclic voltammetry (CV), galvanostatic charge discharge (GCD), and electrochemical impedance spectroscopy (EIS) were carried out using the Autolab, PGSTAT30 Potentiostat-Galvanostat. Electrochemical impedance spectroscopy (EIS), with a frequency range of 100 kHz to 10 mHz, was used for this study, which was done under open circuit potential (OCP).

CV and GCD curves can be used to derive the specific capacity (C_s_, F.g^−1^) of the samples using equations (1) and (2), respectively [20].


(1) $$C_{s}=\frac{1}{2\times m\times v\times \Delta V}\int_{V_{a}}^{V_{c}}i_{\left(V\right)}dV$$

(2) $$C_{s}=\frac{i\times \Delta t}{m\times \Delta V}$$

where *m* (g) is the mass of the electrode material, *i* (A) is the current, *Δt* (s) is the discharge time, *v* (mV.s^−1^) is the potential scan rate, and *ΔV* (V) is the potential sweep window.

Using GCD results in accordance with equations (3) and (4), the energy density (E, Wh.kg^−1^) and power density (P, W.kg^−1^) of the synthesized perovskite oxide supercapacitors can be determined [1,19].


(3) $$E=\frac{1}{2}C_{s}V^{2}$$

(4) $$P=3600\times \frac{E}{\Delta t}$$

where, *v* (V) is the sweep potential window.

## 3. Results and discussion

### 3.1. Structure and morphology

One of the most crucial factors influencing the performance of anion intercalation electrode materials is crystal structure. The distortion of the structure and content of oxygen vacancies has a significant effect on the ion diffusion rate in the perovskite oxide electrode. Therefore, the capacity, electrochemical performance, and cyclic stability of the electrode depend on the perovskite oxide structure [1].

**Figure 1:**
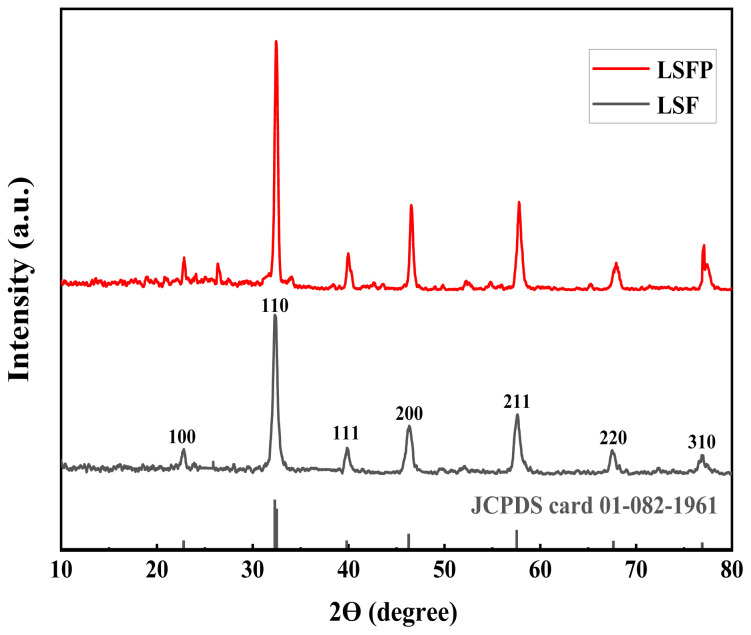
XRD pattern of LSF and LSFP over 10–80° 2θ range.

The phase and crystal structure of the synthesized materials were studied using X-ray diffraction analysis (XRD) in a 2θ range of 10° to 80°. According to Figure 1, the diffraction pattern obtained for the LSF sample corresponds to the La_0.6_Sr_0.4_FeO_3-δ_ (JCPDS card 01-082-1961) pattern. This compound has a cubic structure and belongs to the Pm-3m space group, which does not have any impurity phase. In addition, the diffraction pattern obtained for LSFP is also in accordance with the diffraction pattern obtained in the work of Di Bartolmeo et al. for the La_0.6_Sr_0.4_Fe_0.9_Pd_0.1_O_3-δ_ sample [20]. The diffraction patterns obtained for both samples indicate oxygen vacancy of size δ. It can be clearly seen that the intensity of the diffraction peak increased after the addition of Pd. The observed diffraction peaks for LSFP are slightly shifted to the right relative to the position of the LSF peaks. The significant distortion in the crystal structure of LSFP can explain this phenomenon [4,8]. Pd doping in the B site of the LSF structure induced crystal structure distortion according to the John Teller effect and the Goldschmidt tolerance factor due to the difference in ionic radii of Pd and Fe [21–24]. Therefore, LSFP is expected to have higher oxygen vacancy content than LSF due to higher distortion.

**Figure 2:**
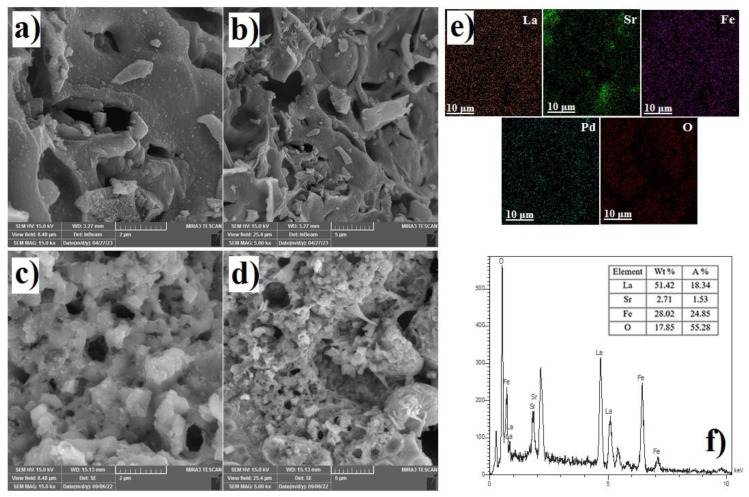
SEM image of a. and b. LSF; c. and d. LSFP; e. elemental patterns of LSFP; f. EDX spectrum of LSF.

The morphology of perovskite oxides was investigated using SEM analysis as shown in Figures 2a–2d. Both perovskite materials had the same morphology and were in condense form. Common perovskite oxides, prepared at high temperatures, have such a morphology [22]. SEM images demonstrate that the Pd-doped perovskite oxide is mostly made up of particles with diameters in the tens of nanometers, which give a larger specific surface area to promote interaction between the electrode’s active sites and the electrolyte. Elemental mapping analysis was used to check the distribution of elements in the samples. Elements (La, Sr, Fe, Pd, and O) were shown in different colors in the results of the elemental mapping of LSFP. As shown in Figure 2e, the distribution of elements in the selected area of the structure is homogeneous, which shows that Pd is correctly placed in the LSF structure. In addition, energy dispersive X-ray (EDX) was also performed for LSF as shown in Figure 2f. The molar ratio of La, Sr, Fe, and O in LSF was about 18.34: 1.53: 24.85: 55.28, which shows that the amount of Pd doping is equal to the theoretical amount and the synthesis of the sample has been done successfully.

**Figure 3:**
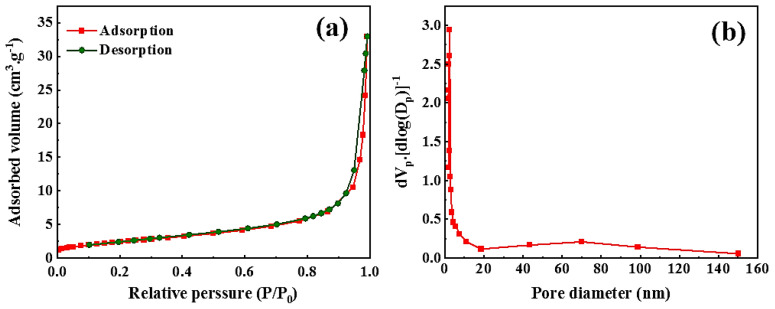
a. N_2_ adsorption-desorption isotherm of LSFP, b. Pore size distribution of LSFP.

The analysis of nitrogen adsorption and desorption measurements was carried out to investigate the specific surface area and the nature of the porosity of the synthesized oxides (Figure 3). The average pore size for LSF and LSFP were found to be 16.54, and 19.71 nm, respectively. Also, the specific area of LSF and LSFP were 7.03 and 8.94 m^2^.g^−1^, respectively. The results showed that LSFP has a larger specific surface compared to other samples, the high specific surface along with excellent active points improves the electrochemical activity of perovskite oxide.

**Figure 4:**
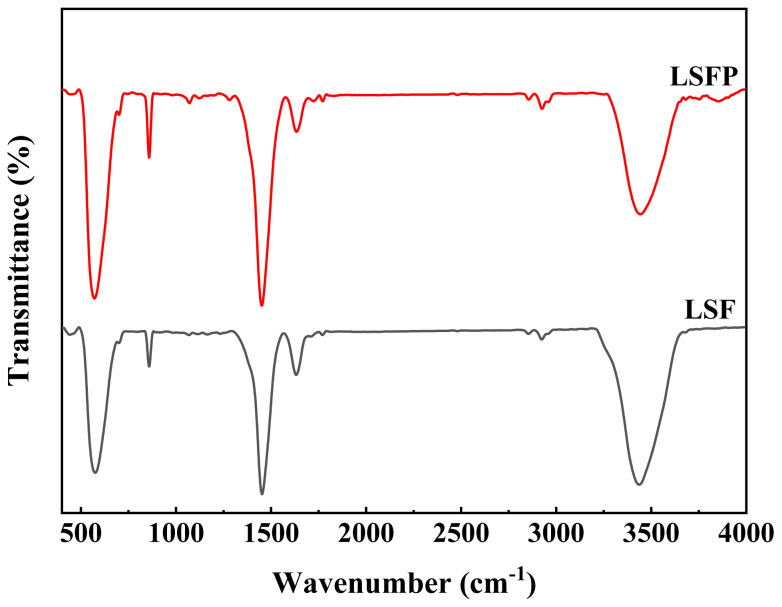
FT-IR spectrum of the LSF and LSFP.

FT-IR spectroscopy is one of the important methods used to investigate the chemical composition of materials. Figure 4 shows the FT-IR spectrum of LSF and LSFP. By comparing the two obtained spectra, a significant similarity can be clearly observed between them. Perovskite oxides usually have a strong peak around 580 cm^−1^, which is related to the asymmetric stretching of the B cation in the BO_6_ octahedral [25]. The B site element is actually a transition metal that may be present in the structure in its various oxidation states. The vibration of different oxidation states of B-site cation can be assigned to the observed peaks around 800–1000 cm^−1^ and 1450–1700 cm^−1^ [26–30]. As can be seen, with Pd doping, the peak intensity in these regions increased for LSFP, which is probably related to the greater distortion of the BO_6_ octahedral in this sample. The broad absorption band that occurred at about 3400 cm^−1^ is related to the stretching of the hydroxyl functional group (O-H) present in adsorbed water on the sample surface. The observed peaks around 2850 and 2920 cm^−1^ related to the symmetric and asymmetric stretching of C-H bonds, respectively [24,28].

**Figure 5:**
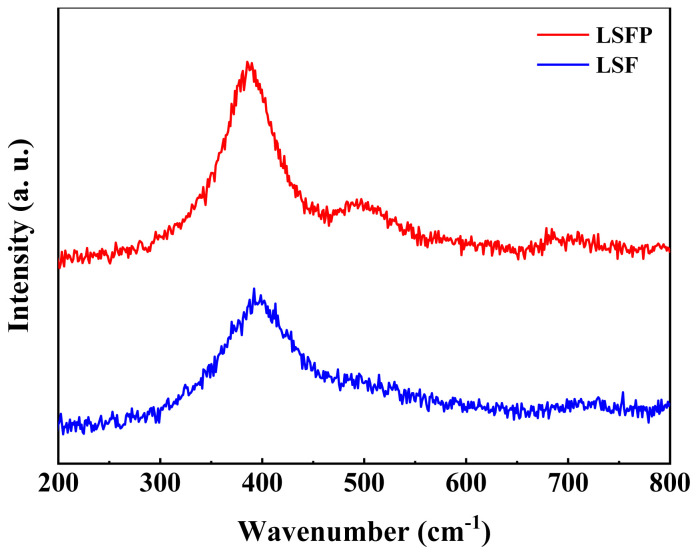
Raman spectra of the LSF and LSFP.

Raman spectroscopy is very useful in investigating the distortion of the structure and content of oxygen vacancies [1]. Therefore, to study the structural properties and mobility of ions and electrons in perovskite oxides, Raman spectroscopy was performed, and the results are presented in Figure 5. The observed Raman peak at about 390 cm^−1^ corresponds to the B_3g_ assigned mode [29]. This shows the bond tension peak related to FeO_6_. As it is evident from the obtained Raman spectra, after Pd doping, the intensity of this peak has increased, which indicates more distortion of the structure.

### 3.2 Electrochemical characterization

The activity and electrochemical performance of perovskite oxides, which are employed as supercapacitor electrodes, are highly dependent on the cations of the B site in the structure. The property of mixed ionic electronic conductivity of perovskite oxides, originates from the octahedral activity of BO_6_, because the cation of the B site is a transition metal and can be in different oxidation states during the process. As a result of the change in the oxidation state of the B site cation, an electrochemical reaction is performed, which is accompanied by the exchange of ions and electrons [1,15]. Electrons of the n-type and holes of p-type perovskites are considered in the place of B^n+^ and B^(n+1)+^ cations, respectively, which can transfer along the B-O-B chain. In addition, perovskite oxides inherently contain oxygen vacancies. These oxygen vacancies act as the starting point for ionic mobility in the perovskite structure and lead to improved ionic conductivity [4]. This observed behavior of perovskite oxides can be changed by making partial substitutions at the A and B sites. The electrochemical activity of the synthesized perovskite oxides was evaluated using CV, GCD, and EIS analyses.

**Figure 6:**
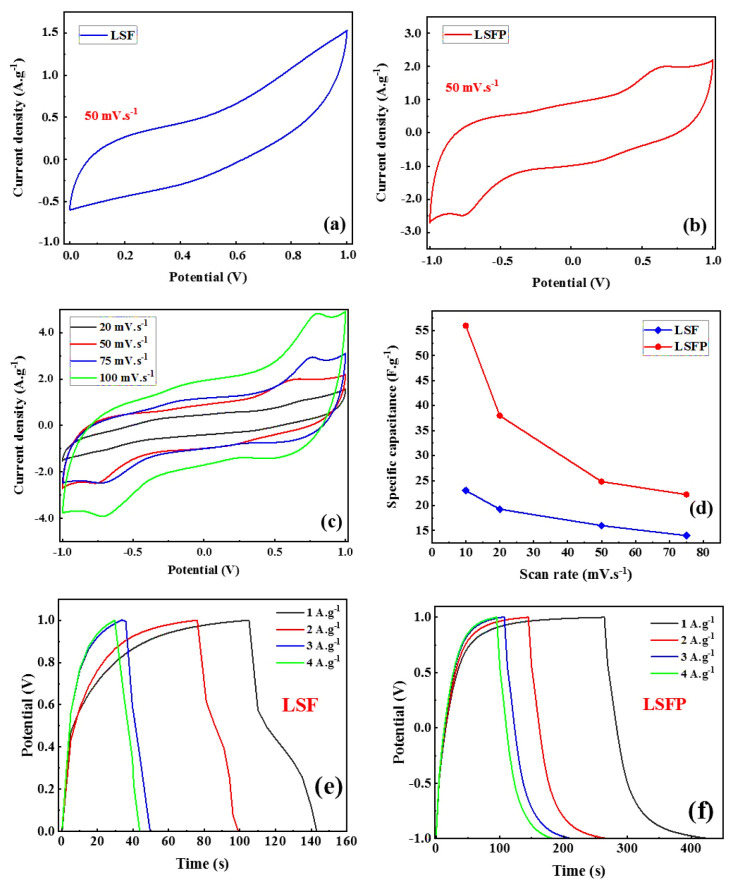
a. and b. CV curves of LSF and LSFP; c. CV curves of LSFP at different scan rates; d. specific capacitance of LSF and LSFP at different scan rates; e. and f. GCD curves of LSF and LSFP.

Using 1M KOH as the electrolyte, CV analysis of synthesized perovskite oxides was carried out in a two-electrode system. Figures 6a and 6b show the obtained CV curves for LSF and LSFP, respectively. As can be seen, the potential window obtained for LSFP is in the interval (−1)–(+1); while, the LSF potential window is located in the range of 0–(+1). A larger potential window is directly related to power and energy density, therefore, LSFP is better than LSF in terms of power and energy density. In addition, two peaks are clearly observed in the CV curve related to LSFP, which is related to the increase of the oxidation states of the B site cation from the B^n+^ state to the B^n+1^ state [8,10,31]. Observing the aforementioned peaks in the CV curve actually shows that the electrochemical reaction in LSFP is much more dynamic than it is in LSF. Additionally, Figure 6c displays the CV curves for LSFP at various scan rates. As can be seen, redox peaks of the CV curve are still evident with increasing scan rate, which indicate that this sample has high active points (oxygen vacancy). Figure 6d also shows how the specific capacitance changes with increasing scan rate for LSF and LSFP. In all scanning rates, the specific capacitance of LSFP was higher than that of LSF, which shows that the partial substitution of Pd in the LSF structure improves its electrochemical activities and imposes more oxygen defects on the LSF structure. The specific capacitance of both samples also falls as the scan rate rises; this behavior may be caused by cation leaching during the charge-discharge process at high scan rates [32,33].

For further study, the galvanostatic charge-discharge (GCD) test for pseudo-capacitors was investigated in 1M KOH at room temperature. Figures 6e and 6f show the GCD curves of LSF and LSFP, respectively, at a current density of 1 A.g^−1^. The curves obtained from the GCD analysis have an asymmetric shape, which denotes the pseudo-capacitance behavior of the synthesized perovskite oxides [8,24]. By comparing the two GCD curves, it is clearly observed that the discharge time of LSFP has increased significantly compared to LSF, which indicates the high specific capacitance of this perovskite oxide. In addition, according to the obtained GCD results, the IR drop of both synthesized samples is about 0.43 V. Therefore, the operating potential related to LSFP and LSF is obtained as 1.53 and 0.53, respectively, which directly affects the power density and energy density according to equations (3) and (4). In this way, the results of GCD are completely consistent with the results of CV. The specific capacitance was obtained at current densities of 1, 2, 3, and 4 A.g^−1^ for LSF 28, 23, 17.1 and 15.2 F.g^−1^, respectively. Also, the values of specific capacitance for LSFP in similar current densities were equal to 80, 62, 54.82, and 46.7 F.g^−1^, respectively. Table 1 presents the numerical values obtained from the GCD curves for the specific capacitance related to LSF and LSFP in comparison with several other works [34,35]. The excellent electrochemical performance of LSFP is related to its structural features, especially the oxygen vacancies of the structure. In fact, by increasing the content of oxygen vacancies in the structure, more active sites are available to the OH^−^ species in the electrolyte, and the electrochemical reaction occurs better and more easily [1,26]. As a result, more energy is saved. Also, an energy density of 44.45 W.h.kg^−1^ was obtained at a power density of 1000 W.kg^−1^ and a current density of 1 A.g^−1^ for LSFP.

**Figure 7:**
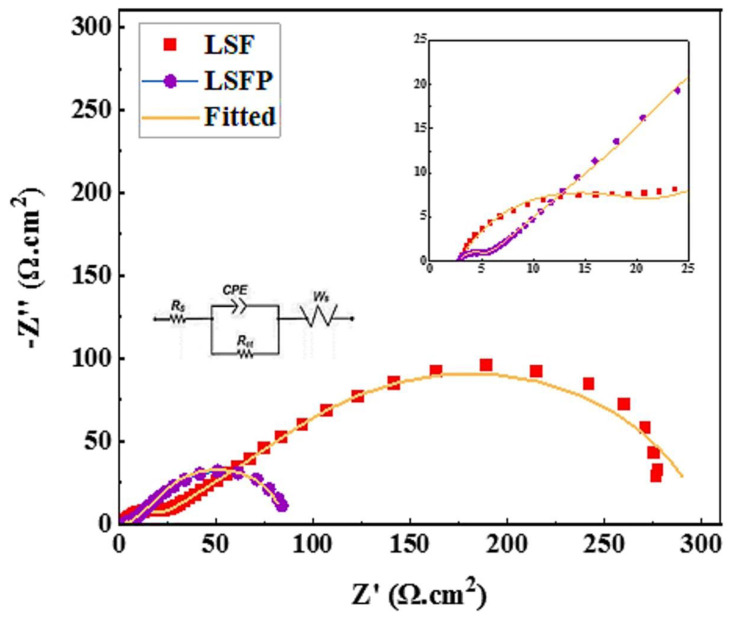
Nyquist plots of LSF and LSFP.

**Figure 8:**
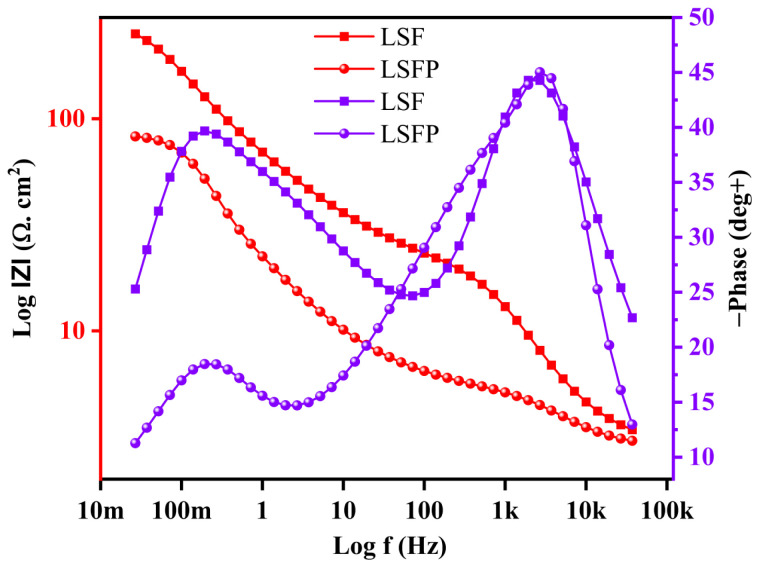
Bode plot of LSF and LSFP.

Electrochemical impedance spectroscopy (EIS) was studied in the open circuit potential (OCP) for all samples. Based on the Nyquist curves of LSF and LSFP as shown in Figure 7, an equivalent circuit was drawn. In this comparable circuit, R_ct_ and R_s_ refer to the charge transfer resistance and the solution resistance, respectively. As can be seen, the ion and electron transfer resistance of LSFP is lower than LSF. Quantitative values obtained from EIS are presented in Table 2. Also, a short Warburg has been observed in the Nyquist curves, which is probably because the ion diffusion layer is limited due to the fact that little electrode and electrolyte materials have been used, and this limitation of the diffusion layer has appeared in the Nyquist diagrams, in the form of a short Warburg at low frequencies [1,28,36]. Figure 8 shows the Bode plot of both synthesized samples.

**Figure 9:**
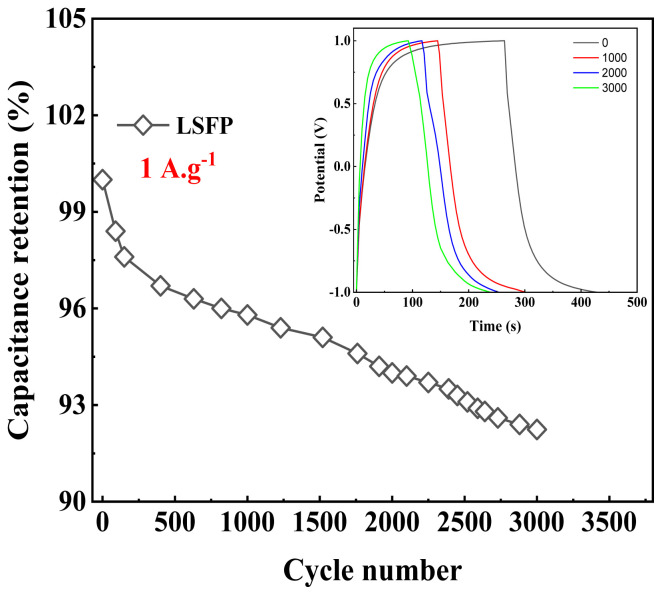
Change of specific capacitance of LSFP with increasing number of cycles. (The inset is GCDs of LSFP at the first, the 1000^th^, the 2000^th^ and the 3000^th^ cycle.)

In addition, by using long charge-discharge cycles, the stability of the LSFP was investigated. From the results of this analysis presented in Figure 9, it is clearly seen that the specific capacitance of LSFP decreases with the increase in the number of cycles. This phenomenon is affected by cation leaching during long charge-discharge cycles [9]. The stability analysis results showed that LSFP can maintain 92% of its initial capacitance after 3000 cycles.

## 4. Conclusions

In this study, perovskite oxides La_0.6_Sr_0.4_FeO_3_ (LSF) and La_0.6_Sr_0.4_Fe_0.9_Pd_0.1_O_3_ (LSFP) were prepared for evaluation as an anion intercalation supercapacitor electrode material using the sol-gel combustion method. The partial substitution of Pd in the structure of the perovskite oxide has increased the oxygen vacancy of the LSF structure, as a result of which its electrochemical performance has been significantly improved. Because the oxygen vacancy sites are actually the active sites of the perovskite oxide surface, where the electrochemical reaction of the supercapacitor is carried out by adsorbing the OH^−^ ions of the electrolyte. The diffusion of O^2−^ ions is also significantly affected by structural oxygen vacancies. The aforementioned electrochemical process on the surface of the perovskite oxide causes the pseudo-capacitive behavior of the synthesized perovskite oxides. At a current density of 1 A.g^−1^, the LSFP’s specific capacitance of 80 F.g^−1^ was measured. Another finding was that a symmetrical LSFP cell has an energy density of 44.45 W.h.kg^−1^ at 1000 W.kg^−1^ of power density and 1 A.g^−1^ of current density.

## Figures and Tables

**Table 1:** The specific capacitance of LSF and LSFP compared to other works.

Perovskite oxides	Potential window (V)	Specific capacitance	Ref.
LSF//LSF	0 – (+1)	28 F.g^−1^ at 1 A.g^−1^	this work
LSFP//LSFP	(−1) – (+1)	80 F.g^−1^ at 1 A.g^−1^	this work
Gd_0.7_Sr_0.3_NiO_3_// Gd_0.7_Sr_0.3_NiO_3_	0 – (+2)	56.7 F.g^−1^ at 1 A.g^−1^	[7]
La_0.85_Sr_0.15_MnO_3_//AC	0 – (+1.2)	19.5 F.g^−1^ at 0.2 A.g^−1^	[8]
SrMn_0.875_Nb_0.125_O_3_//AC	0 – (+2)	84.25 mF.cm^−2^	[16]
La_0.5_Ca_0.5_MnO_3_// La_0.5_Ca_0.5_MnO_3_	0 – (+1.2)	21.7 F.g^−1^ at 0.1 A.g^−1^	[26]
AC//SrCoO_3-δ_	0 – (+1.6)	87 F.g^−1^ at 1 A.g^−1^	[27]
MnO_2_@ SrCo_0.875_Nb_0.125_O_3_@carbon cloth//CuS @ carbon cloth	0 – (+1.8)	114.6 mF.cm^−2^	[34]
CeNiO_3_//CeNiO_3_	0 – (+1.65)	117 C.g^−1^ at 1 A.g^−1^	[35]

**Table 2:** Electrochemical impedance parameters obtained for LSF and LSFP at the frequency ranges of 100 kHz to 10 mHz.

Sample	R_s_ (Ω cm^2^)	CPE		R_ct_ (Ω cm^2^)	W-R (Ω cm^0.5^)	Fitting error
		Y_0_×10^−5^ (Ω^−1^cm^−2^S^n^)	n			
**LSF**	2.57	3.10	0.85	13.78	288.8	0.0038
**LSFP**	2.79	16.01	0.8	2.22	78.96	0.0013
